# The Function of Cortactin in the Clustering of Acetylcholine Receptors at the Vertebrate Neuromuscular Junction

**DOI:** 10.1371/journal.pone.0008478

**Published:** 2009-12-29

**Authors:** Raghavan Madhavan, Zhuolin L. Gong, Jin Jin Ma, Ariel W. S. Chan, H. Benjamin Peng

**Affiliations:** Department of Biology, The Hong Kong University of Science and Technology, Kowloon, Hong Kong, China; Medical College of Georgia, United States of America

## Abstract

**Background:**

Postsynaptic enrichment of acetylcholine receptors (AChRs) at the vertebrate neuromuscular junction (NMJ) depends on the activation of the muscle receptor tyrosine MuSK by neural agrin. Agrin-stimulation of MuSK is known to initiate an intracellular signaling cascade that leads to the clustering of AChRs in an actin polymerization-dependent manner, but the molecular steps which link MuSK activation to AChR aggregation remain incompletely defined.

**Methodology/Principal Findings:**

In this study we used biochemical, cell biological and molecular assays to investigate a possible role in AChR clustering of cortactin, a protein which is a tyrosine kinase substrate and a regulator of F-actin assembly and which has also been previously localized at AChR clustering sites. We report that cortactin was co-enriched at AChR clusters in situ with its target the Arp2/3 complex, which is a key stimulator of actin polymerization in cells. Cortactin was further preferentially tyrosine phosphorylated at AChR clustering sites and treatment of myotubes with agrin significantly enhanced the tyrosine phosphorylation of cortactin. Importantly, forced expression in myotubes of a tyrosine phosphorylation-defective cortactin mutant (but not wild-type cortactin) suppressed agrin-dependent AChR clustering, as did the reduction of endogenous cortactin levels using RNA interference, and introduction of the mutant cortactin into muscle cells potently inhibited synaptic AChR aggregation in response to innervation.

**Conclusion:**

Our results suggest a novel function of phosphorylation-dependent cortactin signaling downstream from agrin/MuSK in facilitating AChR clustering at the developing NMJ.

## Introduction

At the vertebrate neuromuscular junction (NMJ) motor nerve-secreted acetylcholine (ACh) binds and opens postsynaptic ACh receptors (AChRs) to initiate excitation along the muscle membrane and cause contraction. One remarkable feature of the NMJ is its unfailing postsynaptic response to every nerve-stimulation, which is made possible by the selective enrichment of AChRs in muscle within a small membrane domain that apposes presynaptic “active zones” where synaptic vesicles dock and release ACh. Whereas ∼10,000 AChRs are present per µm^2^ of the synaptic muscle membrane, only ∼10 AChRs are found per µm^2^ of the extrasynaptic membrane [1]. Thus, in the assembly of the NMJ, synaptic AChR clustering is a critical and perhaps also the most studied step [2].

During development AChRs form aggregates in embryonic muscle fibers even before motor innervation due to the activation of the muscle receptor tyrosine kinase MuSK [3], [4]. This “pre-patterned” clustering of AChRs in the central regions of muscle fibers involves, in addition to MuSK, the transmembrane protein LRP4 [5] and the adapter dok-7 which enhances MuSK signaling [6]. Next, during innervation, a nerve-deposited heparan-sulfate proteoglycan named agrin [7] stimulates MuSK to promote AChR clustering and stabilization locally at synapses [8], [9]. How agrin activates MuSK has remained unclear because agrin does not bind to MuSK [8], although recent studies suggest that agrin interacts with LRP4 and that LRP4 binds to MuSK and facilitates the aggregation and (trans)activation of MuSK [10], [11]. Finally, as AChRs become accumulated at newly established synapses, the pre-patterned AChR clusters are disassembled, either by synaptogenic stimuli or by ACh [12], [13], [14], which helps the selective concentration of AChRs at NMJs.

How are AChRs concentrated in the postsynaptic membrane? It is thought that AChRs diffusing freely on the muscle surface become clustered at traps generated by synaptogenic stimuli at the NMJ [15]; this diffusion-mediated trapping of AChRs has recently been directly visualized through single-molecular tracking with quantum dots [16], [17]. The clustering of AChRs is mediated by the protein rapsyn, which crosslinks and tethers AChRs to the cortical actin cytoskeleton [2], [12], [13], [18]. F-actin and several proteins that bind to it are enriched at the NMJ and at AChR clusters in muscle cells, and inhibition of actin polymerization blocks the aggregation of AChRs in response to synaptogenic stimulation [19], [20]. Furthermore, AChR clustering involves signaling by Rho-family GTPases [21], which regulate diverse actin polymerization-driven processes [22], p21-activated kinase 1, an effector of the GTPase Cdc42 [23], and geranylgeranyl transferase, an enzyme which enhances the membrane association and activation of GTPases [24]. Conversely, inhibition of PI3 kinase signaling in myotubes reduces agrin-dependent activation of Rac and Cdc42 GTPases and impedes AChR clustering [25].

The above findings suggest that Rho GTPases influence AChR aggregation in multiple ways, but little is known about how the receptor clustering process is affected by other proteins that also regulate actin polymerization. The focus of this study is on one such protein – cortactin – which has been shown to localize at AChR clusters in cultured muscle cells [19], [26], but for which no functional role in NMJ formation has been described to date. Initially identified as a major src tyrosine kinase substrate in cells [27], [28], cortactin is today recognized as a modulator of numerous actin polymerization-dependent processes, ranging from cell motility to endocytosis to dendritic spine growth and synaptogenesis in central neurons [29]. An important target of cortactin is the Arp2/3 protein complex, which binds to existing actin filaments and initiates new branch growth [30], [31]; association of cortactin with the Arp2/3 complex enhances Arp2/3-induced actin polymerization [29], [32]. Intriguingly, phosphorylation by src allows cortactin to be linked by the adapter Nck1 to another Arp2/3-stimulator, N-WASP, and this tripartite complex composed of phospho-cortactin, Nck1 and N-WASP activates Arp2/3-dependent actin polymerization better than cortactin or N-WASP alone [33]. In light of these recent findings related to cortactin and the importance of tyrosine kinase signaling and actin polymerization in AChR clustering, here we asked three specific questions: Is cortactin's tyrosine phosphorylation relevant in the context of AChR clustering in situ? Is cortactin's phosphorylation in muscle affected by agrin/MuSK signaling? Does cortactin signaling in any way regulate synaptic AChR aggregation? The results of our cell biological, biochemical and molecular assays presented below suggest that phosphorylation-dependent signaling by cortactin downstream from agrin/MuSK promotes AChR clustering at the NMJ.

## Results

### Localization of Actin-Polymerization Regulators at AChR Clustering Sites

**Figure 1:**
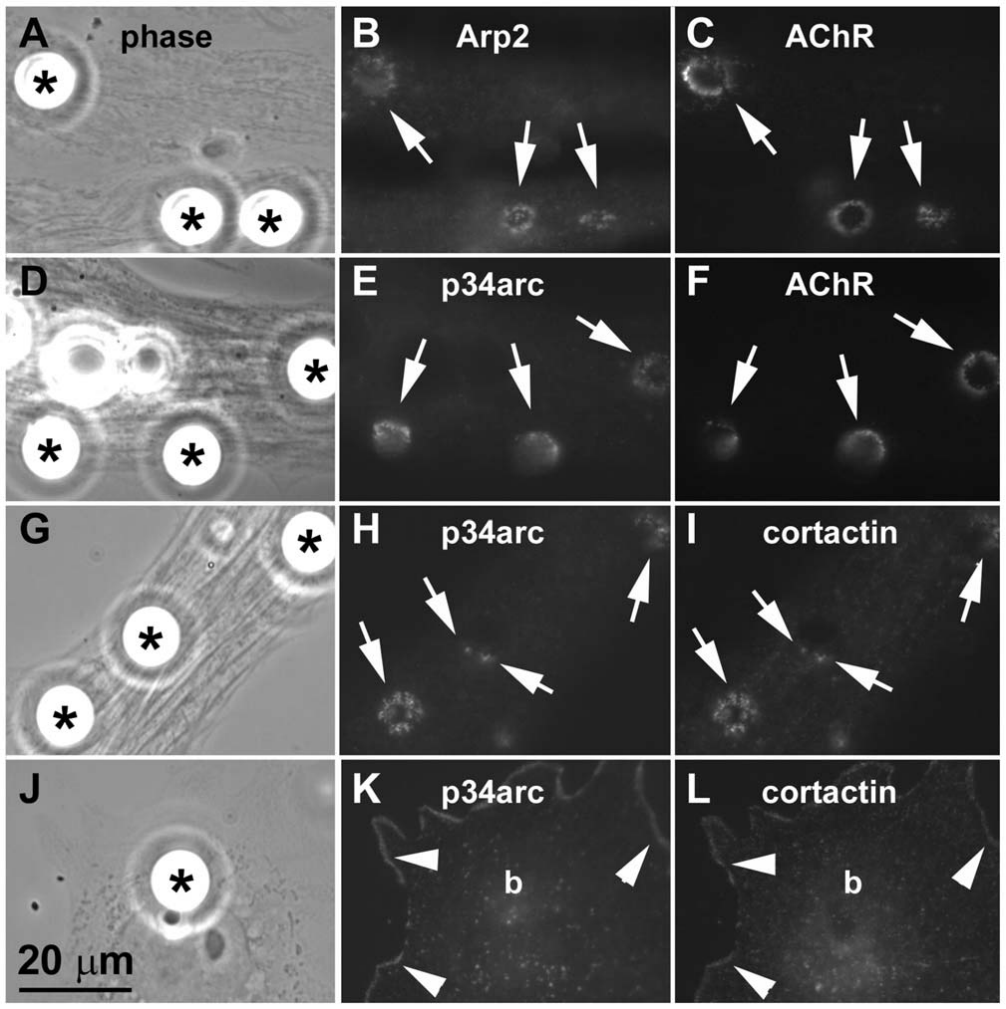
Localization of Arp2/3 complex proteins and cortactin at AChR clustering sites. Cultured embryonic Xenopus muscle cells labeled with rhodamine-α-bungarotoxin (R-BTX) were stimulated overnight with polystyrene beads coated with heparan-binding growth-associated molecule (HB-GAM) (A, D; asterisks) to induce AChR clusters (C, F). Cells were then fixed and labeled with affinity-purified polyclonal antibodies against the Arp2/3 complex proteins Arp2 (B) and p34arc (E) followed by FITC-linked anti-rabbit secondary antibodies. Separately, bead-stimulated muscle cells (G) were labeled with anti-p34arc polyclonal (H) and anti-cortactin monoclonal (I; mAb4F11) antibodies and then FITC-conjugated anti-rabbit and rhodamine-conjugated anti-mouse secondary antibodies. AChRs, Arp2 and p34arc were clustered at bead-muscle contacts (A-F; arrows) where cortactin localized and overlapped in distribution with p34arc (H and I; arrows). In primary muscle cultures non-muscle cells were occasionally found (J) and in these cells p34arc (K) and cortactin (L) localized along the cell periphery (arrowheads) but were not clustered at bead-cell contacts (“b” in K and L corresponds to bead indicated by asterisk in J).

We previously showed that dynamic F-actin assembly occurs at sites of de novo AChR clustering and that green fluorescent protein (GFP)-tagged cortactin is recruited to such sites [19], [20]. Because the Arp2/3 complex is a key cellular regulator of actin polymerization and a target of cortactin, we asked whether proteins of this complex are localized in muscle cells where AChR clustering is stimulated. Primary cultures of Xenopus embryonic muscle cells were labeled with rhodamine-α-bungarotoxin (R-BTX) to mark AChRs and then stimulated with polystyrene beads coated with heparan-binding growth associated molecule (HB-GAM); this procedure enables AChR clusters to be induced reliably and in a spatiotemporally controlled manner [20]. Labeling of bead-treated cells with affinity-purified antibodies revealed that Arp2 and p34arc, two members of the Arp2/3 complex, were enriched at bead-muscle contacts where AChRs were focally clustered (Fig. 1, A–F), but numerous unrelated proteins were not (data not shown). The distribution of the Arp2/3 complex proteins relative to AChRs resembled that of cortactin [26], which can directly bind to the Arp2/3 complex [32], and when bead-treated muscle cells were labeled by the polyclonal anti-p34arc antibody and a monoclonal anti-cortactin antibody, p34arc and cortactin were found to colocalize at bead-muscle contacts (Fig. 1, G–I). However, in non-muscle cells which are found occasionally in primary muscle cultures, p34arc and cortactin were detected along the cell periphery but not at bead-contacts (panels J–K), suggesting that the antibodies against p34arc and cortactin recognized their targets at AChR clusters in muscle cells and did not simply mark all sites where beads contacted cells.

### Association of Tyrosine-Phosphorylated Cortactin with AChR Clusters

**Figure 2:**
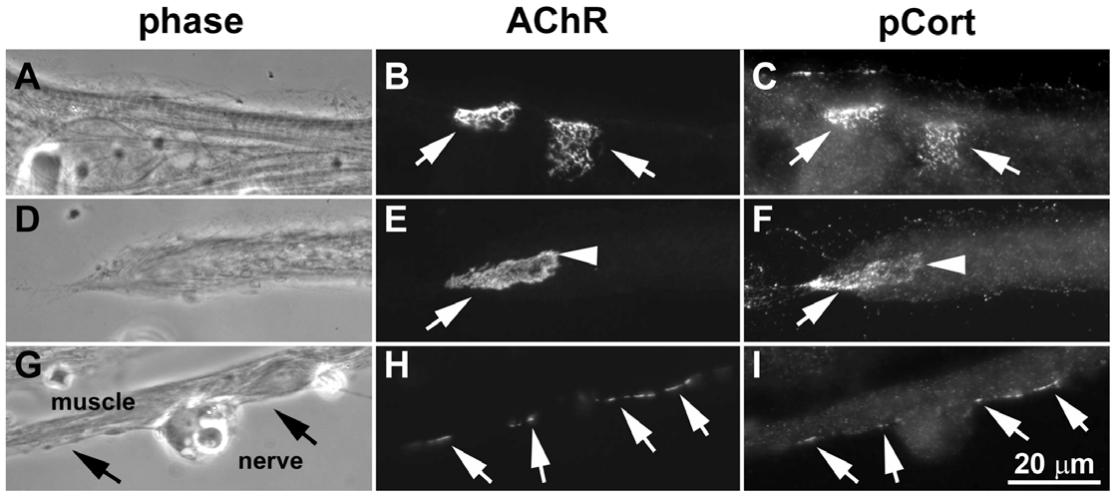
Tyrosine phosphorylation of cortactin at pre-patterned and nerve-induced AChR clusters. Xenopus muscle cells were labeled with R-BTX and after fixation with an antibody that specifically recognizes Y482-phospho-cortactin (plus FITC-linked anti-rabbit antibodies) (A-F). In some cases muscle cells were first co-cultured for 1 d with spinal neurons and then labeled with R-BTX and anti-phospho-cortactin and secondary antibodies (G-I). In pure muscle cultures (A, D) large “pre-patterned” AChR clusters were present (B, E; arrows) and at these sites staining by anti-phospho-cortactin was significantly stronger than elsewhere in muscle cells (C, F; arrows). Labeling for phospho-cortactin was detected at almost all pre-patterned clusters examined (see Table S1), although within the clusters certain regions at times appeared to be more enriched in phospho-cortactin than others (as in F; arrow versus arrowhead). The anti-phospho-cortactin antibody also labeled muscle cell edges (C, F) where cortactin is known to be localized. In nerve-muscle co-cultures (G) AChRs were selectively concentrated at synaptic contacts (H; arrows) and these nerve-induced AChR clusters were also labeled by the anti-phospho-cortactin antibody (I; arrows).

Because recent work has shown that tyrosine phosphorylation of cortactin's src-target sites enhances the activation of Arp2/3-dependent actin polymerization [32], [33], we tested whether cortactin phosphorylation is relevant in the context of AChR clustering. For this the distribution of tyrosine-phosphorylated cortactin relative to AChR clusters was examined in Xenopus muscle cells by labeling (separately) with three different antibodies directed against cortactin phosphorylated on its major src-target sites (Y421, Y466 and Y482 in mouse cortactin). In quiescent muscle cells anti-Y482-phospho-cortactin antibody strongly labeled pre-patterned AChR clusters (Fig. 2, A–F) and also the edges of cells (where cortactin is known to localize) but only weakly labeled other regions of the cells. Y482-phosphorylated cortactin was detected reliably at pre-patterned AChR clusters (panel C), although sometimes it appeared more concentrated in certain regions of the clusters than others (panel F). Data from several muscle culture preparations showed phosphorylation of Y482-cortactin at >95% of pre-patterned clusters identified (Table S1, Supporting Documents).

Antibodies against Y421- and Y466-phospho-cortactin also labeled AChRs clusters and muscle cell edges, but with these two antibodies staining was somewhat weaker than with anti-Y482-phospho-cortactin (not shown). It is possible that these commercial antibodies have differing affinities for their target sites or that these sites are phosphorylated and/or accessible to different extents. Unlike these three antibodies, however, many control antibodies showed markedly different labeling of muscle cells. For example, a polyclonal antibody against tyrosine-phosphorylated AChR β-subunit labeled only AChR clusters, two different polyclonal antibodies against cadherin complex proteins strongly stained cell junctions but not AChR clusters, and other polyclonal antibodies either labeled muscle cells uniformly (such as an anti-phospho-Shp2 phosphatase antibody) or failed to label the cells (such as anti-synapsin) (R.M., A.W.S.C., H.B.P., unpublished observations).

**Figure 3:**
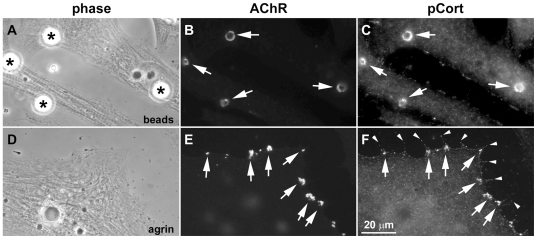
Cortactin phosphorylation at AChR clusters induced by growth factor-coated beads and agrin. R-BTX-labeled Xenopus muscle cells were stimulated overnight with HB-GAM-beads (A-C) or neural agrin (D-F). In cells exposed to beads (A; asterisks) AChRs aggregated at bead-muscle contacts (B; arrows) and strong labeling was detected at these bead-induced AChR clusters for Y482-phospho-cortactin (C; arrows). Treatment of muscle cells with agrin (D) generated numerous small (∼0.5-3 µm) AChR clusters (D; arrows) and antibody labeling showed that phospho-cortactin was enriched at these clusters (E; arrows) and also along myopodia that formed near the AChR clusters (F; arrows and arrowheads).

Next we examined whether cortactin is phosphorylated at sites of new AChR cluster formation. In Xenopus nerve-muscle co-cultures, AChR clusters developed in muscle cells focally at sites where the cells were contacted by nerves and these clusters were strongly labeled by the anti-Y482-phospho-cortactin antibody (Fig. 2, G–I). Phospho-cortactin's localization closely matched that of postsynaptic AChR clusters, but labeling for phospho-cortactin was weak in presynaptic neurites (which were strongly labeled, as expected, by antibodies against neuronal markers such as synapsin; not shown). From several nerve-muscle co-culture preparations we found phospho-cortactin at >80% of nerve-induced AChR clusters (Table S1). Moreover, in muscle cultures stimulated with HB-GAM-beads, phospho-cortactin was found to be enriched at AChR clusters induced at bead-muscle contacts (Fig. 3, A–C), with pooled data showing that this was the case at ∼95% of such clusters (Table S1), and in agrin-treated muscle cells phospho-cortactin was accumulated at new AChR clusters (∼0.5–3 µm in diameter) generated across the muscle surface (Fig. 3, D–F). Interestingly, phospho-cortactin was also detected along myopodia (panel D–F) generated close to AChR clusters [34] and was often concentrated at myopodial tips (arrowheads in panel F). Taken together these results suggested that phosphorylation of cortactin's src-target tyrosines is enhanced at sites in muscle where synaptogenic stimuli such as agrin produce their known functional effects.

### Agrin-Dependent Tyrosine Phosphorylation of Cortactin

**Figure 4:**
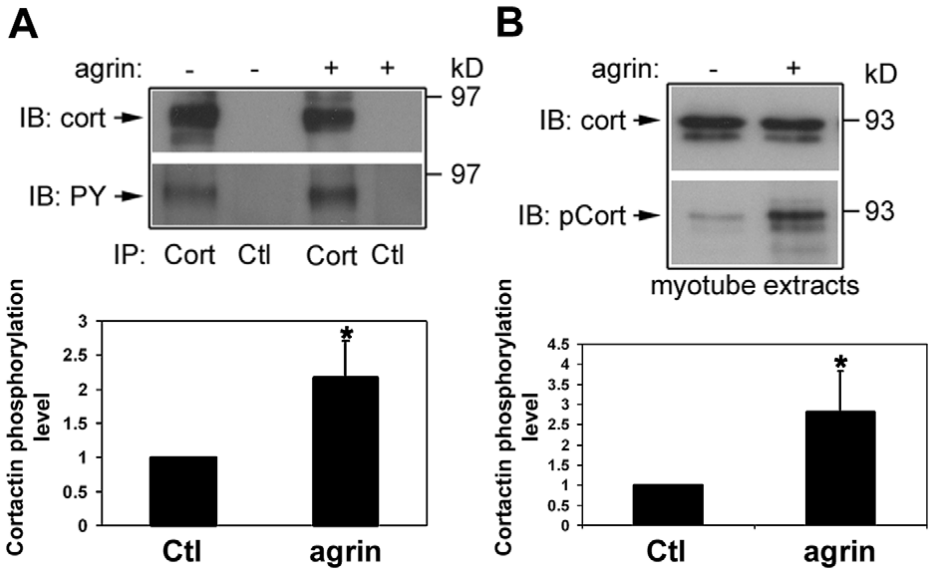
Agrin-dependent enhancement of cortactin tyrosine phosphorylation. Cultured C2 mouse myotubes were exposed to medium without (-) or with added agrin (+) before preparing extracts for immuno-precipitation (A) with a monoclonal antibody against cortactin (IP: cort) or an unrelated protein (IP: ctl). When these samples were immuno-blotted for cortactin (IB: cort) and total phosphotyrosine (IB: PY; mAb4G10), cortactin was found to be captured only by the anti-cortactin antibody (upper lanes), and anti-phosphotyrosine staining showed that cortactin from extracts of agrin-treated cells was tyrosine phosphorylated significantly more than that captured from control extracts (lower lanes). This increase in cortactin phosphorylation was quantified from four experiments (A, graph) by measuring band densities, normalizing for cortactin loading (see Methods), and calculating the phosphotyrosine level change relative to control. B. To test whether the src-target sites in cortactin were phosphorylated in response to agrin-treatment, myotube extracts were blotted with antibodies against total cortactin and cortactin phosphorylated on Y421. Agrin-treatment did not alter the amount of cortactin present in extracts (upper lanes) but the staining of cortactin by the anti-Y421-phospho-cortactin antibody (IB: pCort) was enhanced by agrin-treatment more than two-fold, as shown by quantification from three experiments (B, graph). Positions of pre-stained MW markers (Bio-Rad) are indicated on the right side of blots, and in the graphs * represents P<0.02 in t-tests.

To extend the studies described above we tested whether agrin signaling directly affects cortactin's tyrosine phosphorylation through biochemical assays. For this C2 mouse myotubes were used because such assays cannot be easily performed on the limited material provided by Xenopus embryonic muscle primary cultures. Fully differentiated C2 myotubes were exposed to control or agrin-containing medium for 1 h and then cortactin was immuno-precipitated from them for analyses. Blotting of immuno-precipitates with anti-cortactin and anti-phosphotyrosine antibodies (Fig. 4A, top) showed that the anti-cortactin antibody (mAb4F11) captured cortactin from extracts but the control antibody did not (“IB:cort”, upper blot). Significantly, cortactin immuno-precipitated from extracts of agrin-treated myotubes was more strongly stained by the anti-phosphotyrosine antibody than cortactin captured from extracts of control myotubes (“IB: PY”, lower blot). From four such experiments we determined the band intensities of cortactin stained by anti-cortactin and anti-phosphotyrosine and then divided the latter values by the former to normalize for cortactin loading in each case. These results indicated that cortactin's tyrosine phosphorylation level in agrin-treated myotubes was more than twice that in control myotubes (Fig. 4A, bottom).

As discussed in the previous section, three major src-target sites have been identified in cortactin. To test whether agrin-treatment of muscle, which activates src signaling [35], triggers the phosphorylation of these specific sites, C2 myotube extracts were immuno-blotted with anti-cortactin and anti-Y421-phospho-cortactin antibodies. Both antibodies stained bands of cortactin's expected size and anti-cortactin staining showed equal protein loading (Fig. 4B); with the anti-phospho-cortactin antibody, however, cortactin was stained nearly three-times more strongly in extracts of agrin-treated myotubes than of control myotubes (Fig. 4B, bottom; data from three experiments). Similarly, a band corresponding to full-length cortactin was detected with the anti-Y466-phospho-cortactin antibody and this was enhanced by agrin as well (not shown), but staining by the anti-Y482-phospho-cortactin antibody in total extracts was weak and we have been unable to ascertain whether that staining is altered significantly by agrin-treatment. Nevertheless, these findings and our in situ labeling results together supported the conclusion that phosphorylation of cortactin's src-target sites is enhanced during agrin/MuSK signaling.

### Involvement of Cortactin Signaling in Agrin/Nerve-Induced AChR Clustering

To investigate whether phosphorylation-dependent cortactin signaling is involved in the AChR clustering process, we expressed a dominant-negative, phosphorylation-defective mutant cortactin and wild-type cortactin in C2 myotubes and examined agrin-induction of AChR clusters. In the mutant cortactin (3YF-cortactin), the three major src phosphorylation sites – Y421, Y466 and Y482 – were eliminated [36], and both the mutant and the wild-type cortactin proteins were tagged with GFP. The GFP-tagged cortactin proteins (or GFP alone) were expressed in myotubes by mRNA transfection, which was carried out in parallel on myotubes grown on coverslips for analysis of AChR clustering and in culture dishes for biochemically confirming the expression of the exogenous proteins.

**Figure 5:**
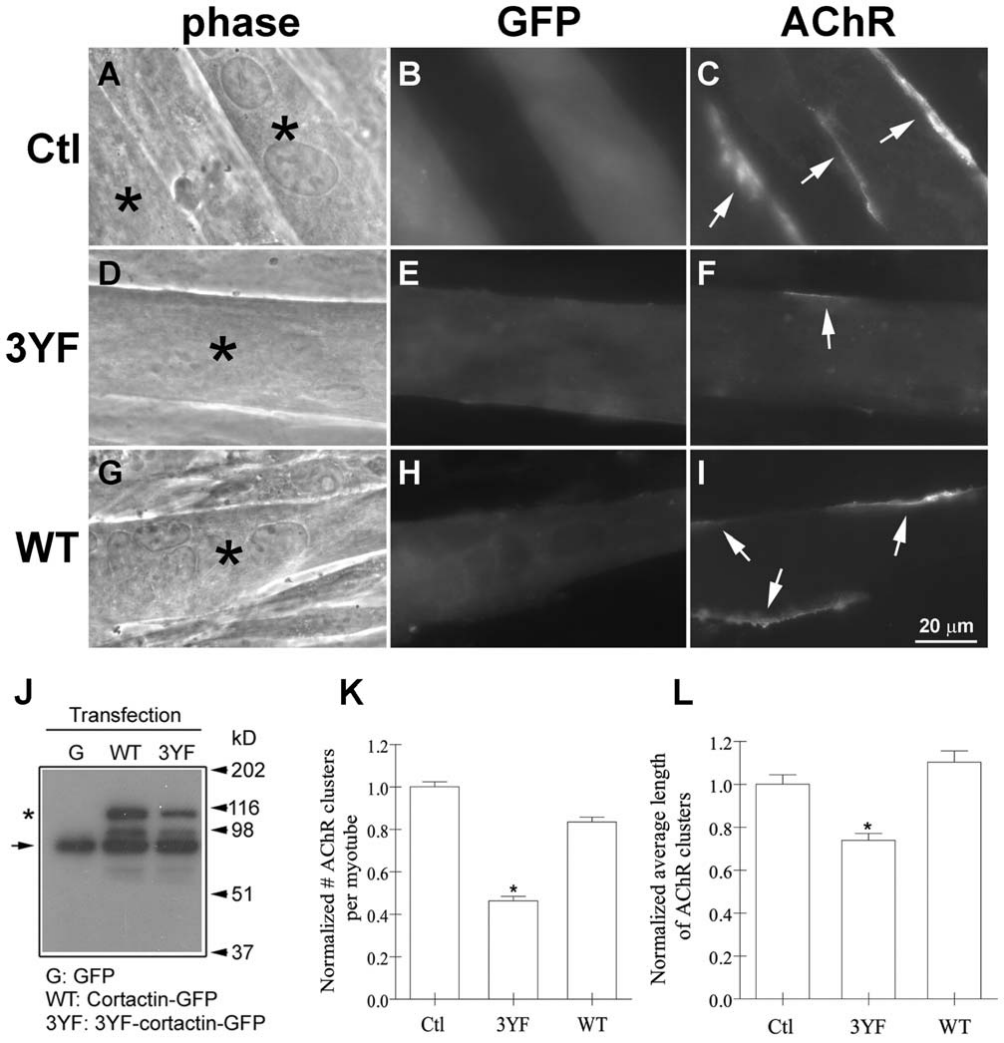
Inhibition of agrin-induced AChR clustering by forced expression of phospho-mutant cortactin in myotubes. To examine the effect of exogenous cortactin proteins on AChR clustering, C2 myotubes were transfected with mRNAs encoding GFP (Ctl) or GFP-tagged phospho-mutant (3YF) cortactin or wild-type (WT) cortactin. After treating myotubes with agrin overnight, cells expressing exogenous proteins (A, D, G; asterisks) were identified by green fluorescence (B, E, H) and the AChR clusters present on the surface of these cells were examined by R-BTX-labeling (C, F, I; arrows). Forced expression of the phospho-mutant, but not wild-type, cortactin reduced the number and lengths of agrin-induced AChR clusters in myotubes. J. To biochemically confirm the expression of exogenous cortactin proteins in myotubes, extracts prepared from myotubes transfected with mRNAs encoding GFP, GFP-tagged WT and 3YF cortactin were immuno-blotted with anti-cortactin monoclonal antibody mAb4F11. Myotubes transfected with GFP mRNA (G) contained full-length endogenous cortactin (arrow on left), but those transfected with WT- and 3YF-cortactin mRNAs contained endogenous cortactin plus a protein (∼25 kD larger) corresponding to exogenous, GFP-tagged cortactin (asterisk). MW marker positions are indicated on the right. K-L. Myotubes transfected with GFP or GFP-tagged cortactin proteins were selected randomly and the numbers and lengths of the AChR clusters present on their surface were determined; data from five separate transfection experiments were pooled and normalized relative to values obtained from GFP-tranfected cells. Fewer (K) and smaller (L) AChR clusters were present in myotubes expressing phospho-mutant cortactin than in cells expressing GFP alone or WT-cortactin-GFP. Mean and SEM values are shown, *P<0.05.

Myotubes growing on coverslips were incubated overnight with agrin and labeled with R-BTX (Fig. 5) and transfected cells were identified by green fluorescence. In these we found that compared to myotubes expressing GFP (panels A–C) or wild-type cortactin-GFP (panels G–I), those expressing the phospho-mutant cortactin had fewer and smaller AChR clusters (panels D–F). Immuno-blotting of extracts of myotubes transfected with mRNAs encoding the GFP-tagged cortactin proteins (but not GFP) demonstrated that anti-cortactin monoclonal antibody 4F11 stained endogenous cortactin plus the exogenous cortactin proteins, which were ∼25 kD larger (Fig. 5J). From five separate transfection experiments the numbers and lengths of AChR clusters in green fluorescent (transfected) myotubes were determined; for this the myotubes were examined in their entirety and all distinct clusters (such as those indicated by arrows in Fig. 5) were counted and measured. Data pooled from 200 or more (each) GFP-cells, wild-type cortactin-cells and phospho-mutant cortactin-cells showed that relative to control (GFP) myotubes, the phospho-mutant cortactin-expressing myotubes had nearly 60% fewer clusters (4.3/myotube instead of ∼10/myotube) which were nearly 30% smaller (∼19 µm long compared to ∼27 µm) (Fig. 5, K–L).

**Figure 6:**
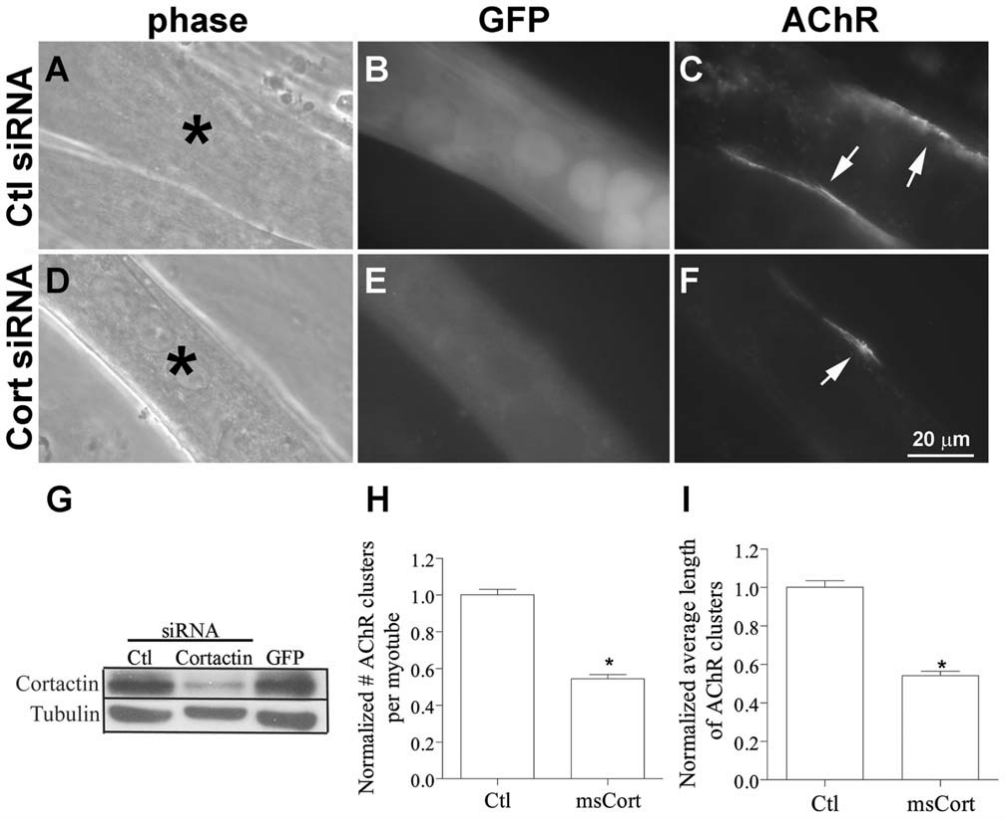
Inhibition of agrin-induced AChR clustering by down-regulation of cortactin expression in myotubes. C2 myotubes generated from myoblasts transfected with control siRNAs (A-C) or a pool of siRNAs directed against mouse cortactin (D-F) (both mixed with a cDNA encoding GFP) were incubated overnight in differentiation medium containing agrin before labeling with R-BTX. Transfected myotubes (A, D; asterisks) were identified by green fluorescence (B, E), and the AChR clusters present on their surface (C, F; arrows) were counted and the lengths of these clusters were measured. G. To demonstrate that siRNAs against cortactin knocked down cortactin expression, in each experiment extracts were prepared from myotubes generated from myoblasts transfected in parallel and maintained under conditions identical to those used for examining agrin-induced AChR clustering. Extracts of cells transfected with GFP cDNA plus control (p120ctn) siRNA (Ctl; left lane), cortactin siRNA (middle lane) or GFP cDNA alone (right lane) were immuno-blotted with antibodies against cortactin (upper blot) or tubulin (lower blot). The cortactin siRNA suppressed the expression of cortactin without affecting unrelated proteins (such as tubulin, which is also shown here to demonstrate equal protein loading), and cortactin's expression was not affected by control siRNAs or by transfection procedures (where only GFP cDNA was used). From four transfection experiments AChR cluster data from control (Ctl) and mouse cortactin (msCort) siRNA-transfected myotubes were pooled and normalized relative to those obtained from cells transfected with the control siRNA. These results showed that agrin-induced AChR cluster numbers (H) and lengths (I) were significantly lower in myotubes expressing reduced levels of endogenous cortactin compared to those expressing normal levels of cortactin. Mean and SEM values are shown, *P<0.05.

The above results demonstrated that agrin-stimulated AChR clustering in myotubes was inhibited in the presence of exogenous phospho-mutant cortactin. Therefore, to directly test whether endogenous cortactin functions in the AChR cluster assembly process, we down-regulated its expression in myotubes using RNA interference (RNAi). C2 myoblasts were transfected with GFP cDNA mixed with a cocktail of mouse cortactin-specific small interfering RNAs (siRNAs) or siRNAs against unrelated proteins; cells were then allowed to differentiate for 4 d before they were treated overnight with agrin. When GFP-positive cells were examined after labeling with R-BTX, the results showed that AChR clustering in response to agrin was impaired in cortactin-siRNA-transfected myotubes compared to control-siRNA-transfected myotubes (Fig. 6, A–F). Immuno-blotting of total cell extracts from the same batch of myotubes (in each experiment; see Methods) confirmed that the cortactin-siRNAs down-regulated the expression of cortactin but did not produce any noticeable change in the expression of many unrelated proteins (Shp2, SIRPα1, p120 catenin (p120ctn), Arp2, Arp3, Nck1 and tubulin; not shown, except tubulin in panel G) and that control siRNAs (against Shp2, SIRPα1 and p120ctn; p120ctn-siRNA used as control in panel G) did not affect cortactin levels. AChR cluster numbers and lengths in control siRNA- and cortactin siRNA-transfected myotubes (150 or more each) were measured (see above) and data from four separate siRNA-transfection experiments revealed that the average values of these parameters were reduced by ∼40% in myotubes expressing lower than normal levels of cortactin (Fig. 6, H–I).

**Figure 7:**
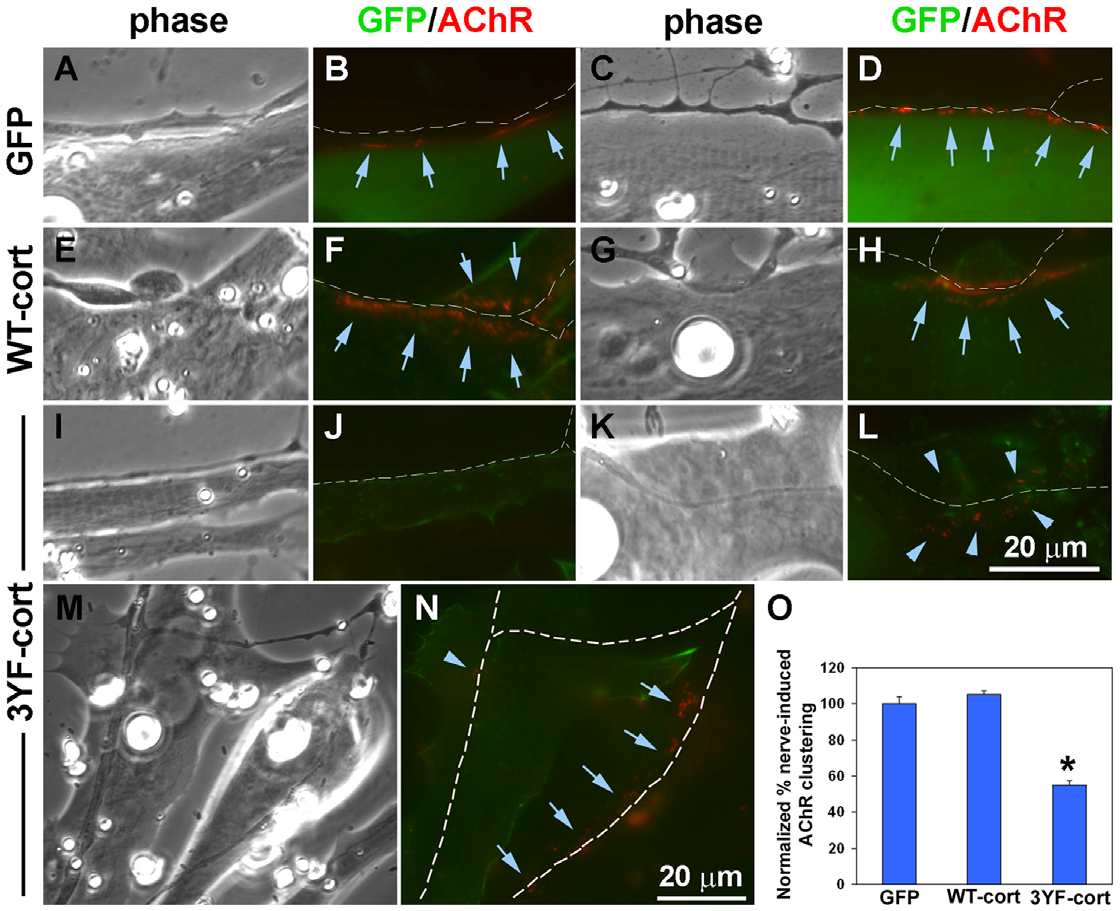
Suppression of synaptic AChR aggregation by phospho-mutant cortactin expressed selectively in muscle cells. Xenopus embryonic muscle cells expressing GFP (A-D) and GFP-tagged wild-type cortactin (WT-cort; E-H) and phospho-mutant cortactin (3YF-cort; I-N) were co-cultured with spinals neurons for 1 d and then labeled with R-BTX to visualize AChR clusters. Cells expressing the exogenous proteins fluoresced green and AChR clusters appeared red, as shown in this figure with 2-3 representative examples of nerves contacting muscle cells with GFP or GFP-tagged cortactin proteins. In the GFP and WT-cort muscle cells (A-H), AChRs were tightly clustered (arrows) along nerve-contacts identified (traced in white in colored panels) but this was not the case in 3YF-cort cells where nerves often induced no AChR clustering (J) or induced few clusters that were loosely organized (L; arrowheads). We found cases where the same neurites moved across normal muscle cells and 3YF-cells (M-N) and in such cases synaptic AChR clustering was robust in the normal cells (arrows) but not mutant cells (arrowheads). O. The percentages of nerve-contacts with AChR clusters were determined by examining several co-cultures with muscle cells expressing GFP or the GFP-tagged cortactin proteins (see Methods) and these values were normalized relative to numbers obtained from examining nerve-contacts on GFP-cells. In muscle cells expressing phospho-mutant cortactin, synaptic AChR clustering was almost halved. Nerve-muscle contacts examined: GFP cells, 168; WT-cort cells, 214; 3YF-cort cells, 225; mean and SEM shown, *P<0.0001.

Is cortactin signaling involved in AChR clustering at the NMJ in vivo? To answer this, we injected mRNAs encoding GFP or GFP-tagged wild-type and mutant cortactin proteins into Xenopus embryos with the goal of examining NMJs that can be observed (in a “chevron” pattern) in the developing tail muscle. These experiments, however, failed to reveal whether cortactin functions in AChR aggregation because the embryos injected with mutant cortactin mRNA died before reaching a stage (∼40 or later) when NMJs can be reliably identified by BTX-labeling. We therefore adopted an alternative approach for testing cortactin's function, an approach that has been successfully used by us [34] and by others [23] in which myotomal muscle cells and spinal neurons are isolated from early embryos (∼stage 20–22) and plated together. In these nerve-muscle co-cultures AChR clustering is triggered in response to innervation, and when nerve and muscle cells isolated from normal and mRNA-injected embryos are mixed together, nerve or muscle can be manipulated selectively with exogenous molecules (Methods). In this experimental system NMJ formation can be observed and quantified by R-BTX-labeling, which is used to monitor the focal aggregation of AChRs at nerve-muscle contacts [34]. When we examined contacts between normal spinal neurons and muscle cells expressing GFP or the GFP-tagged cortactin proteins (Fig. 7), robust AChR clustering was detected at innervation sites in muscle cells expressing GFP (panels A–D) or GFP-tagged wild-type cortactin (E–H); in contrast, in cells expressing GFP-tagged phospho-mutant cortactin, often no synaptic AChR clustering was detected (I–J) or only weak and loosely organized AChR clusters were found (K–L). We also observed cases in which the same neurites contacted both mutant cortactin-expressing cells and normal cells present side-by-side, and here we found that AChR clustering was strongly induced in the normal cells but not mutant cells (M–N). In cells expressing GFP, wild-type and mutant cortactin we identified 168–225 nerve-contacts from multiple culture preparations and classified these innervation sites as those with or without AChR clusters; the quantified results showed that in mutant cortactin cells, taking into account even loosely organized AChR clusters (as in L), nerve-induction of AChR aggregation was only half as effective as that in GFP and wild-type cortactin cells (Fig. 7O).

## Discussion

**Figure 8:**
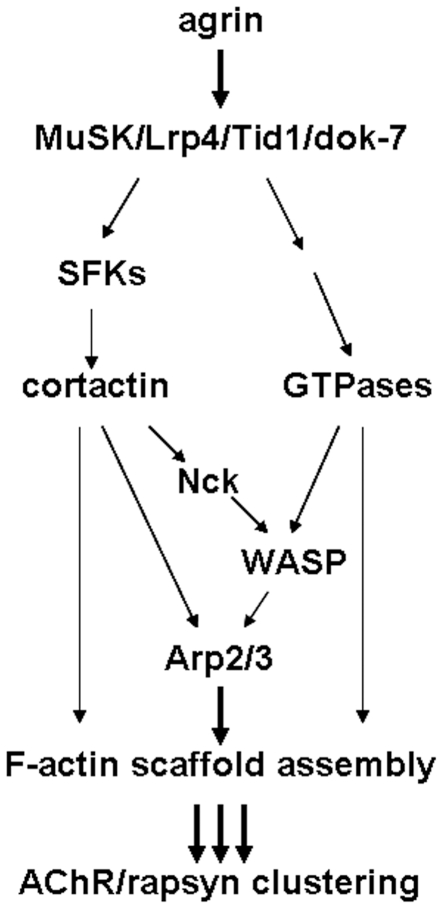
Cortactin signaling in agrin-dependent AChR clustering: a model. Activation of MuSK by agrin induces AChR clustering in an actin polymerization-dependent manner. This model depicts a possible way in which cortactin signaling might promote the AChR clustering process. Initiation of intracellular signaling by the activated MuSK complex could enhance cortactin's tyrosine phosphorylation through src family tyrosine kinases (SFKs) (and possibly other kinases such as abl), and cortactin, in turn, could increase actin polymerization. Alternatively, cortactin might trigger actin polymerization by activating the Arp2/3 complex, either on its own or in concert with WASP-related proteins (N-WASP, WIP, etc.) to which it could be linked by the adapter Nck. In parallel, via other signaling intermediates, MuSK could stimulate Rho-family GTPases and, through them, F-actin assembly. Such enhanced and dynamic actin polymerization at synaptic sites could generate a scaffold which “traps” AChRs through rapsyn.

In this study examining the molecular regulation of AChR aggregation at the NMJ we obtained these novel results: one, proteins of the Arp2/3 complex and cortactin (which can together promote actin polymerization) were co-distributed at AChR clustering sites (where F-actin assembly occurs). Two, AChR clusters were enriched in cortactin phosphorylated on its src-target tyrosine residues, whose phosphorylation enhances Arp2/3-mediated actin polymerization [33]. Three, treatment of myotubes with agrin increased the phosphorylation of cortactin on tyrosine residues, including those known to be targeted by src. Four, forced expression in myotubes of a cortactin mutant lacking these (three major) src-target sites inhibited agrin-induced AChR clustering, as did the depletion of endogenous cortactin in myotubes using RNAi. And five, disruption of normal cortactin signaling in muscle cells by expression of the dominant-negative cortactin mutant inhibited the aggregation of AChRs at innervation sites. We propose that agrin/MuSK signaling acts on cortactin through src (and possibly other) tyrosine kinases, and that cortactin, on its own or with proteins such as Nck1, N-WASP and components of the Arp2/3 complex, promotes actin polymerization and AChR clustering at the NMJ (Fig. 8).

Diverse cellular mechanisms collaborate to generate and maintain high-density AChR aggregates at the NMJ. Muscle-surface AChRs are selectively clustered in the synaptic region of muscle, the clustered AChRs are metabolically stabilized, and AChRs and many proteins that promote their synaptic accumulation are synthesized at high levels in the sub-synaptic domains of myotubes [2], [12], [13], [37]. The first of these mechanisms – synaptic AChR clustering – has arguably been studied most extensively and current evidence supports the following (simplified) view of the molecular pathway involved: agrin secreted by nerve activates MuSK through the protein Lrp4 [10], [11], and MuSK, together with binding partners dok-7 [6] and Tid1 [38], triggers downstream signaling through intracellular Ca^2+^, various kinases and phosphatases and Rho-family GTPases to direct AChR aggregation in an actin polymerization-dependent manner [12], [39].

New F-actin synthesis occurs at AChR clustering sites in muscle cells [19], [20] and likely depends on the combined activity of Rho GTPases and their regulators and effectors that function in agrin/MuSK signaling [21], [23], [24], [25]. Rho GTPases are potent stimulators of the Arp2/3 complex, which enhances actin polymerization in many types of cells [31]. Arp2/3 complex-dependent actin polymerization is activated by proteins such as WASP/N-WASP and Scar/WAVE, and the binding of Rac and Cdc42 GTPases enables WASP/N-WASP and Scar/WAVE to stimulate the Arp2/3 complex [30], [31]. Thus, our detection of Arp2/3 complex proteins at AChR clusters suggests that GTPase signaling accelerates Arp2/3-dependent F-actin assembly at synapses (Fig. 8).

In this study expression of either mutant cortactin or cortactin siRNAs in muscle cells inhibited agrin/nerve-dependent AChR aggregation partially rather than fully. Although this could have been due to incomplete disruption of normal cortactin function in the muscle cells, it could also be because of cortactin being one of many Arp2/3 complex/actin-regulators (such as the GTPases) at the synapse. Our observation that over-expression of wild-type cortactin in myotubes did not enhance AChR clustering is consistent with the latter view. It should be of interest to investigate in future studies the extent to which the inhibition of both cortactin and Rho-family GTPases blocks agrin-induced AChR clustering.

We have shown here that cortactin was colocalized with the Arp2/3 complex at AChR clustering sites. Notably, tyrosine phosphorylated-cortactin was enriched at AChR clusters and the phosphorylation of cortactin's src-target sites was enhanced by agrin treatment of muscle cells. Although cortactin was originally identified as a major src kinase-substrate in cells, the influence of phosphorylation on cortactin-dependent regulation of actin polymerization long remained unclear [29], [40]. An elegant biochemical study carried out recently, however, has indicated a novel mechanism by which phosphorylation of cortactin's src-target sites can stimulate F-actin assembly [33]. Using purified proteins this study showed that phosphorylation of cortactin by src enables the formation of a complex made up of cortactin and the SH2 domain-containing adapter Nck1, plus N-WASP or the WASP-interacting protein, WIP. Consistent with earlier work [41] cortactin stimulated Arp2/3-dependent polymerization of actin by its N-terminal acidic region; this activity was enhanced following src-phosphorylation of cortactin, which promoted Nck1-binding and through Nck1 the recruitment of WIP [33]. After src-phosphorylation cortactin could also be linked by Nck1 to N-WASP, and this complex (through the “VCA” domain of N-WASP) more robustly stimulated F-actin assembly by Arp2/3 than cortactin or N-WASP alone. Importantly, a phospho-mutant (3YF) cortactin was used to demonstrate that src-dependent enhancement of cortactin's ability to stimulate Arp2/3 (through Nck1 and N-WASP or WIP) requires the Y421, Y466 and Y482 sites of cortactin [33]. Forced expression of the same 3YF-mutant cortactin in myotubes, or the suppression of cortactin expression using RNAi, inhibited agrin-induced AChR clustering in this study. It is known that src kinases are rapidly activated by agrin-stimulation of MuSK and that they phosphorylate MuSK, AChRs and associated proteins [35]. Moreover, like phospho-cortactin (this study), activated-src kinases are enriched at AChR clustering sites in situ [12]. Thus src-cortactin signaling may be spatially restricted to the synaptic regions of muscle where intimate coupling of MuSK and src activation could lead to rapid tyrosine phosphorylation of cortactin; this, in turn, could locally enhance Arp2/3-dependent actin polymerization to generate “traps” that capture mobile AChRs.

Currently a role of cortactin at the NMJ in vivo cannot be directly tested because suitable models – viable animals with global or muscle-specific cortactin gene deletion – are unavailable. Moreover, through our own work we noted that Xenopus embryos which had been injected with the phospho-mutant cortactin mRNA failed to grow to a stage at which NMJs in the developing tail could be studied. We were, however, successful in using this dominant-negative approach to show that interference with phosphorylation-dependent cortactin signaling in muscle is sufficient for inhibiting nerve-induced AChR clustering, and by extension NMJ formation, in culture. Interestingly, others have studied mice lacking src kinase activity in muscle and have shown that while NMJs can develop in the muscles of these mice, the synaptic AChR aggregates found are less stable than their counterparts in the muscles of normal mice [42]. One explanation for this observation is that src binds to AChRs and phosphorylates them, which enhances the cytoskeletal linkage of AChRs through rapsyn [43], [44], [45], [46]. The results of this study raise the additional possibility that in the absence of normal src activity, sub-optimal signaling by cortactin leads to the assembly of synaptic scaffolds which hold AChRs poorly at NMJs.

Properly balanced src signaling is important for normal AChR clustering at the NMJ [47], but what all targets of src influence AChR clustering at the NMJ is incompletely understood. By uncovering a role of the src-substrate cortactin in AChR clustering this study suggests a previously unappreciated way by which src could regulate AChR aggregation at the NMJ. Interestingly, two other src-substrates we previously identified as targets in agrin/MuSK signaling produced effects distinct from those described here for cortactin: p120ctn promoted myopodial induction in response to agrin [34], whereas signal-regulatory protein α1 (SIRPα1) stimulated the tyrosine phosphatase Shp2 to limit MuSK-dependent AChR clustering [48] and to also facilitate the dispersal of pre-patterned AChR clusters by synaptogenic stimuli [14]. Intriguingly, cortactin can participate in p120ctn signaling in epithelial cells [49] and can act together with the p120ctn-relative δ-catenin in generating filopodia-like dendritic protrusions in neurons [50]. Moreover, cortactin itself also affects dendritic spine morphogenesis and remodeling, with the depletion of cortactin in neurons causing a reduction in spines [51], [52]. Thus, cortactin-dependent actin polymerization triggered by src could potentially facilitate p120ctn-mediated myopodial assembly at the NMJ as well as synapse formation in central neurons. And, although it is not known which phosphatases dephosphorylate cortactin in muscle cells, the spread of phosphatase activity initiated by agrin/MuSK [12], [14], [53] may bring about the dephosphorylation of extrasynaptic cortactin to maintain efficient actin polymerization by src-cortactin signaling selectively at developing NMJs where agrin stimulates MuSK. Future studies investigating the spatiotemporal control of cortactin's phosphorylation levels during neuronal synapse development and testing whether that phosphorylation affects cortactin's ability to influence the formation of structures such as dendritic spines should yield further insights into cortactin's functions at CNS synapses and elsewhere.

## Methods

### Reagents

Agrin was obtained from the conditioned medium of HEK293 cells transfected with a plasmid encoding neural agrin [54]. Recombinant heparin-binding growth-associated molecule (HB-GAM) was generously provided by Dr. Heikki Rauvala (University of Helsinki). Na-pervanadate, a potent tyrosine phosphatase inhibitor, was prepared by mixing 10 mM Na-orthovanadate with 1.7% hydrogen peroxide in a 50∶1 ratio just before use and diluting this solution as needed [53]. These reagents were purchased: rhodamine-conjugated α-bungarotoxin (R-BTX) (Molecular Probes; Eugene, OR, USA); monoclonal antibodies against cortactin (4F11) and phosphotyrosine (4G10) and a rabbit polyclonal antibody against p34arc (Upstate Biotechnology; Lake Placid, NY, USA); rabbit polyclonal antibodies against cortactin phosphorylated on Y421, Y466 or Y482 (Cell Signaling Technology; Danvers, MA, USA); rabbit polyclonal antibodies against Arp2 and Y390-phosphorylated AChR β-subunit (Santa Cruz Biotechnology; Santa Cruz, CA, USA); monoclonal anti-Shp2 and anti-neurexin-1 antibodies (BD Biosciences; San Jose, CA, USA); monoclonal anti-α-tubulin antibody DM1A (Sigma; St Louis, MO, USA); rhodamine- and FITC-conjugated secondary antibodies (Zymed; South San Francisco, CA, USA); horseradish-peroxidase (HRP)-conjugated secondary antibodies (Jackson Immuno Research Laboratories; West Grove, PA, USA); and Triton X-100 (TX-100) and West Pico enhanced chemiluminescence (ECL) reagent (Pierce; Rockford, IL, USA).

### Cell Cultures

Primary cultures of Xenopus myotomal muscle cells were prepared from stage 20–22 embryos as described previously [55] in accordance with HKUST's established animal handling and care procedures. Muscle cells were plated on glass coverslips coated with entactin-collagen-laminin substrate (Upstate Biotechnology) and used within one week. For some experiments neural tubes of stage 20–22 embryos were dissociated and spinal neurons were seeded on muscle cells plated 3–5 d earlier; these nerve-muscle co-cultures were examined 1 d later [34]. C2 mouse myotube cultures were prepared by growing myoblasts (purchased from ATCC, Manassas, VA, USA) on glass coverslips or in culture dishes in DMEM containing 20% fetal bovine serum (growth medium) until confluence and then inducing differentiation by changing the medium to DMEM containing 2% horse serum (differentiation medium); fresh differentiation medium was added each day for 4–5 d [53].

### Agrin-Treatment, Cell Extract Preparation, Immuno-Precipitation/Blotting

Differentiated C2 myotubes in culture dishes were incubated for 1 h in fresh differentiation medium without or with added agrin (plus 10 µM Na-pervanadate). After rinsing with cold phosphate buffered saline (PBS), extracts were prepared using a TX-100 buffer (100 mM Tris, pH 7.4, 150 mM NaCl, 5 mM EDTA, 1% TX-100, 1 mM Na-pervanadate) [53], with 1 ml buffer being added to a 10 cm culture dish of cells. To clarified extracts anti-cortactin or control antibodies and Protein A/G agarose beads (Santa Cruz Biotechnology) were added, and after mixing for 2 h at room temp the beads were spun down, washed extensively with Tris-buffered saline containing 0.1% TX-100 and then mixed with SDS-electrophoresis sample buffer to elute immuno-precipitated proteins. Samples were electrophoresed, transferred to PVDF membranes and probed with primary antibodies and HRP-linked secondary antibodies for ECL-based detection.

### Synthesis of mRNAs, Myotube Transfection

Wild-type and phospho-mutant mouse cortactin cDNAs were gifts from Drs. Tom Parsons (University of Virginia) and Xi Zhan (University of Maryland), respectively. In the phospho-mutant cortactin (3YF) construct, three src target sites – Y421, Y466 and Y482 – were changed to non-phosphorylatable Fs [36]. Both wild-type and mutant cortactin proteins were tagged with green fluorescent protein (GFP) by inserting the cortactin cDNAs into pEGFP-N1 plasmid (Clonetech); the cortactin-GFP sequences were subcloned into pCS2+ vector [19] and then into pcDNA3.1. The cortactin-GFP encoding plasmids and pCS2+ vector with an insert encoding only GFP were linearized and used for mRNA preparation with the mMessage mMachine mRNA synthesis kit from Ambion, Inc. (Austin, TX, USA). The mRNAs were diluted with Opti-MEM I medium (GIBCO) before using Lipofectamine 2000 (Invitrogen) to transfect them into myotubes that had been maintained in differentiation medium for 4 d; 1 µg mRNA was used per 3.5 cm dish of cells [48]. Myotubes were incubated in transfection solutions for 6 h and then transferred back to differentiation medium before use in experiments.

### Cortactin Knockdown by RNA Interference

A pool of four small interfering RNAs (siRNAs) targeting mouse cortactin and several other siRNAs against unrelated proteins were purchased from Dharmacon RNA Technologies (Lafayette, CO, USA); the siRNAs (100 pmol) were mixed with GFP cDNA (1 µg) and used for transfecting C2 myoblasts (with Lipofectamine 2000) grown in 3.5 cm culture dishes or on glass coverslips placed within the dishes. Transfection was carried out (for 6 h) when cells were ∼60% confluent, and the cells were then put back in growth medium to allow them to reach confluence before transferring them to differentiation medium [53]. After 4 d myotubes were treated overnight with agrin and used for examining total cortactin expression (by immuno-blotting extracts prepared from myotubes growing in dishes) as well as GFP fluorescence and AChR clustering (after R-BTX labeling of cells growing on coverslips placed in the same dishes).

### Co-Culturing of Xenopus Neurons with Muscle Cells Expressing Exogenous Proteins

To express GFP-tagged wild-type and mutant cortactin (or GFP) selectively in muscle cells, mRNAs encoding these proteins were injected into one cell of 2-4-cell stage Xenopus embryos; myotomal muscle cells were cultured from the embryos after they had developed to stage 20–22 [34]. Muscle cells were maintained for 3–5 d and then spinal neurons from normal (uninjected) embryos were seeded on them and allowed to spread for 1 d.

### Labeling, Microscopy and Quantification of AChR Clustering

AChRs on Xenopus muscle cells were labeled with R-BTX either before exposing cells to control medium or medium containing agrin or HB-GAM beads, or after innervation in co-culture experiments. To localize Arp2/3 complex proteins, phospho-cortactin, etc., cells were fixed with cold ethanol, blocked with PBS containing bovine serum albumin, and labeled with primary and fluorescent-secondary antibodies. Cells were examined with a 63X, 1.4 n.a. lens using an Olympus IX70 inverted microscope, equipped with a Hamamatsu ORCAII cooled-CCD camera controlled by MetaMorph software (Universal Imaging, West Chester, PA, USA).

To analyze AChR clustering in C2 myotubes, cells were treated with agrin overnight, labeled with R-BTX, fixed with 4% paraformaldehyde and then mounted on slides. All transfection assays were carried out multiple times with duplicate or triplicate samples and AChR clusters were examined in (only) transfected (green fluorescent) myotubes. After selecting transfected myotubes randomly, all AChR clusters on those myotubes were counted and their lengths were measured. Average numbers of AChR clusters per myotube and the average lengths of clusters were calculated and normalized relative to values obtained for clusters from cells expressing GFP only.

In Xenopus nerve-muscle co-cultures, effects of exogenous proteins on synaptic AChR clustering was quantified as follows: in R-BTX-labeled live cultures, nerve-muscle pairs were randomly identified (in phase-contrast) in which nerves directly contacted muscle cells; then, if the muscle cell was found to be green fluorescent (indicating the expression of GFP- or GFP-tagged cortactin proteins), we determined (using the rhodamine filter) whether or not AChRs were concentrated focally at nerve-muscle contacts. Co-cultures were prepared using muscle cells from several separate batches of mRNA-injected embryos and neurons from normal embryos and in each preparation 3–6 coverslips were examined. The number of nerve-muscle contacts and the number of contacts with AChR clusters were determined and the latter values were divided by the former; pooled data were normalized using numbers obtained from co-cultures in which muscle cells only expressed GFP.

## Supporting Information

Table S1 Association of phospho-cortactin with AChR clusters.
(0.03 MB DOC)
