# Correction: The Function of Cortactin in the Clustering of Acetylcholine Receptors at the Vertebrate Neuromuscular Junction

**DOI:** 10.1371/annotation/7df19997-b7c7-40a2-9ed9-dc31233ce8f6

**Published:** 2010-02-17

**Authors:** Raghavan Madhavan, Zhuolin L. Gong, Jin Jin Ma, Ariel W. S. Chan, H. Benjamin Peng

The first two authors, Raghavan Madhavan and Zhuolin L. Gong, should be noted as contributing equally to this work. 

