# Supplementary material for: The Function of Cortactin in the Clustering of Acetylcholine Receptors at the Vertebrate Neuromuscular Junction
Source: PLoS One. 2009 Dec 29;4(12):e8478. doi: 10.1371/journal.pone.0008478 (PMC2793544; doi:10.1371/journal.pone.0008478)
Supplement: Table S1 — Association of phospho-cortactin with AChR clusters. (0.03 MB DOC) [file pone.0008478.s001.doc]

**Supporting Document**

**Table S1. Association of phospho-cortactin with AChR clusters**

|  | # AChR clusters | % AChR clusters with phospho-cortactin | # muscle cells examined |
| --- | --- | --- | --- |
| Pre-patterned | 184 | 95.8 ± 0.98 | 111 |
| Nerve-induced | 70 | 86.63 ± 4.65 | 70 |
| Bead-induced | 485 | 94.83 ± 2.14 | 127 |

AChR clusters in control and nerve/bead-stimulated Xenopus muscle cells were examined for labeling by an antibody against Y482-phosphorylated cortactin. In control muscle cells AChR clusters developed spontaneously (were “pre-pattered”), whereas in muscle cells co-cultured with spinal neurons or stimulated with growth factor-coated beads AChR clusters were induced at nerve/bead-muscle contacts. Phospho-cortactin was considered to be associated with only those induced AChR clusters where labeling for it was selectively and significantly elevated, as shown in the representative examples in Figs. 2 and 3.
